# Flavokawain A Induces Apoptosis in MCF-7 and MDA-MB231 and Inhibits the Metastatic Process *In Vitro*

**DOI:** 10.1371/journal.pone.0105244

**Published:** 2014-10-06

**Authors:** Nadiah Abu, M. Nadeem Akhtar, Swee Keong Yeap, Kian Lam Lim, Wan Yong Ho, Aimi Jamil Zulfadli, Abdul Rahman Omar, Mohd Roslan Sulaiman, Mohd Puad Abdullah, Noorjahan Banu Alitheen

**Affiliations:** 1 Bright Sparks Unit, Universiti Malaya, Kuala Lumpur, Malaysia; 2 Faculty of Biotechnology and Bimolecular Sciences, Universiti Putra Malaysia, Serdang, Selangor Darul Ehsan, Malaysia; 3 Faculty of Industrial Sciences & Technology, Universiti Malaysia Pahang, Kuantan, Pahang, Malaysia; 4 Institute of Bioscience, Universiti Putra Malaysia, Serdang, Selangor Darul Ehsan, Malaysia; 5 Faculty of Medicine and Health Sciences, Universiti Tunku Abdul Rahman, Cheras, Selangor, Malaysia; 6 School of Biomedical Sciences, University of Nottingham Malaysia Campus, Semenyih, Selangor, Malaysia; 7 Faculty of Medicine and Health Sciences, Universiti Putra Malaysia, Serdang, Selangor Darul Ehsan, Malaysia; University of Alabama at Birmingham, United States of America

## Abstract

**Introduction:**

The kava-kava plant (*Piper methsyticum*) is traditionally known as the pacific elixir by the pacific islanders for its role in a wide range of biological activities. The extract of the roots of this plant contains a variety of interesting molecules including Flavokawain A and this molecule is known to have anti-cancer properties. Breast cancer is still one of the leading diagnosed cancers in women today. The metastatic process is also very pertinent in the progression of tumorigenesis.

**Methods:**

MCF-7 and MDA-MB231 cells were treated with several concentrations of FKA. The apoptotic analysis was done through the MTT assay, BrdU assay, Annexin V analysis, cell cycle analysis, JC-1 mitochondrial dye, AO/PI dual staining, caspase 8/9 fluorometric assay, quantitative real time PCR and western blot. For the metastatic assays, the *in vitro* scratch assay, trans-well migration/invasion assay, HUVEC tube formation assay, *ex vivo* rat aortic ring assay, quantitative real time PCR and western blot were employed.

**Results:**

We have investigated the effects of FKA on the apoptotic and metastatic process in two breast cancer cell lines. FKA induces apoptosis in both MCF-7 and MDA-MB231 in a dose dependent manner through the intrinsic mitochondrial pathway. Additionally, FKA selectively induces a G2/M arrest in the cell cycle machinery of MDA-MB231 and G1 arrest in MCF-7. This suggests that FKA's anti-cancer activity is dependent on the p53 status. Moreover, FKA also halted the migration and invasion process in MDA-MB231. The similar effects can be seen in the inhibition of the angiogenesis process as well.

**Conclusions:**

FKA managed to induce apoptosis and inhibit the metastatic process in two breast cancer cell lines, *in vitro*. Overall, FKA may serve as a promising candidate in the search of a new anti-cancer drug especially in halting the metastatic process but further *in vivo* evidence is needed.

## Introduction

Breast cancer is among the leading cause of cancer-related deaths among women today [1]. It is estimated that in 1 out of 16 women will be diagnosed with breast cancer at some point in their lives [1]. Conventional treatments include chemotherapy and surgery, nevertheless, these methods have several drawbacks including physical pain, increased relapse and lower survival rate [2], [3]. One of the main reasons administering anti-cancer agents in cancer patients is to eliminate cancer cells; it is also favorable that it inhibits the metastatic process as well. [4]. Natural products have played an important part in search for new drugs, even some of the most famous widely used drugs are derived from natural sources [5], [6].

Kava-kava plant is an evergreen shrub that is widely consumed in the pacific region [7], [8]. This plant is largely known to be involved in a wide spectrum of biological activities including, anti-inflammation, anti-bacterial and most importantly, anti-cancer [9], [10]. Intriguingly, there has been a correlation between the consumption of kava-kava and the incidence of cancer [11]. There are several interesting components that can be found in the kava root extracts, including chalcones [7], [10]. Chalcones are open ring flavonoids that are widely synthesized in the plant kingdom [12]. Flavokawain A is a chalcone and has been reported to possess promising anti-cancer and anti-inflammatory activities [10]. Additionally, flavokawain A was found to inhibit the growth of bladder cancer cell lines *in vitro* [10].Nevertheless, the effects of flavokawain A in breast cancer cells *in vitro*, as well as the anti-metastatic activity have not been studied yet. Based on the preliminary study, flavokawain A was found to have similar potential cytotoxic activities in breast cancer as to bladder cancer cells. Since breast cancer has become the most diagnosed type of cancer in women, especially in Malaysia and generally, worldwide, we aim to investigate the *in vitro* cytotoxic and anti-metastatic effects of flavokawain A on two types of breast cancer cell lines, MCF-7 and MDA-MB231.Both MCF-7 and MDA-MB231 are well-established breast cancer cell lines but they differ in several aspects including, the p53 status, estrogen receptor status and invasiveness [13]. Therefore we could assess the effectiveness of flavokawain A in a more wholly approach.

## Materials and Methods

### Synthesis of flavokawain A

Flavokawain A was synthesized by Claisen-Schmidt condensation reaction of the corresponding acetophenone (1.5 mmole) and 4-methoxybenzaldehyde (1.25 mmole). Both acetophenone and aldehyde were mixed together in the presence of 40% solution KOH.The reaction mixtured was stirred for 18 hrs at room temeprature. The product was finally purified by using flash column chromatography using ethylacetate:hexane of 1∶1 as eluent. Flavokawain A was then recrystallized from MeOH to yield yellow crystalline flat compound. Finally the FKA was characterized by spectroscopic techniques such as IR, UV, EI-MS and NMR data analyses.

### Cell Culture

The cell lines MCF-7, MDA-MB231 and MCF-10A were obtained from the ATCC collection (ATCC, USA). MCF-7 cells were maintained in RPMI, while MDA-MB231 cells were maintained in DMEM. Both media were supplemented with 10% fetal bovine serum and 1% penicillin-streptomycin. MCF-10A cells on the other hand, was maintained in DMEM-F12 media supplemented with hydrocortisone (0.5 µg/ml), insulin (10 µg/ml), hEGF (20 ng/ml) and 10% (v/v) FBS. All the cells were kept in a 37°C incubator equipped with 5% CO2.

### MTT Assay

The MTT assay was conducted in accordance to Mosmann (1983) with slight modifications [14]. The cells were seeded in 96-well plates at a concentration of 0.8x10^5^ cells/well. The cells were then incubated in a 37°C CO_2_ incubator overnight. The following day, flavokawain A was added to the wells with seven different concentrations. The cell viability was measured at 72 hours post-treatment. MTT solution (5 mg/ml) was added at a volume of 20 µl in each well and was incubated for three hours. Afterwards, the solution was discarded, and 100 µl of DMSO was added to each well to solubilize the crystals. The plates were then read using an ELISA plate reader at the wavelength of 570 nm (Bio-tek instruments, USA). Triplicates were carried out for each cell line. The following formula was used to determine the percentage of viable cells.
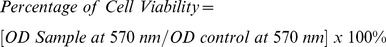



### Cell Treatment

Based on the results of the MTT assay, three doses of flavokawain A were used in the remaining assays. The doses are the IC_25_, IC_50_ and IC_75_ values of flavokawain A when administered to MCF-7 and MDA-MB231. The values are according to Table 1.Since flavokawain A is not soluble in water, this compound was dissolved in DMSO and the volume of DMSO administered to cells was below 0.1%.

**Table 1:** The values of IC_25_, IC_50_ and IC_75_of FKA in MCF-7 and MDA-MB231 to be used in other assays.

FKA Concentrations	IC_25_ (µM)	IC_50_(µM)	IC_75_(µM)
MCF-7	9	25	70
MDA-MB231	6.5	17.5	65

### BrdU Proliferation Assay

The BrdU cell proliferation assay was conducted according to the manufacturers protocol. The cells were cultured in 96-well plates at a density of 0.8x10^4^ cells/well overnight. The following day the cells were treated with three different concentrations of flavokawain A. After the designated hours of treatment, 24 hours prior, the cells were labeled with BrdU and tagged with the anti-BrdU antibody. Afterwards the substrate was added and the colored product was measured using the µquant ELISA plate reader (Bio-tek Instruments, USA) at 450 nm.

### AO/PI Double Staining

AO/PI double staining was assayed to determine the morphological changes of MCF-7 and MDA-MB231 upon treatment with flavokawain A. The cells were seeded in 6 well plates at a concentration of 2.4x10^5^ cells/well and were incubated overnight. The next day, the cells were treated with three different concentrations of FKA, IC_25_, IC_50_ and IC_75_ for 48 h and 72 hours. Afterwards, the cells were trypsinized and washed with PBS twice. The harvested cells were resuspended in 100 µl PBS and stained with 10 µg/ml of each dye. Acridine Orange is a stain that is permeable to viable cells and can stain the cell's DNA directly [15]. This dye emits a green fluorescence once it is excited [15]. Propidium Iodide on the other hand, is a dye that is impermeable to viable cells [15]. It can bind to DNA only when the cells are dead; it emits a red-orange fluorescence instead [15]. The mixture was viewed under a fluorescent microscope (Nikon, Japan).

### Cell Cycle Analysis

The cells were seeded in 6 well plates at a density of 2.4x10^5^ cells/well. The following day, the cells were treated with three different concentrations of flavokawain A. After 12 hours and 24 hours, the cells were trypsinized, washed with PBS and collected. The resulting pellet was fixed in 70% ethanol and stored at -20°C. After a week, the fixed cells were washed with PBS and treated with Rnase and Triton-x, and were then stained with PI (Sigma, USA). Afterwards, the cells were subjected to flow cytometric analysis using the FACS Calibur flow cytometer (Becton Dickinson, USA).

### Annexin V/FITC Assay

The Annexin V assay was carried out using the Annexin V Kit (BD Pharmingen, USA). The cells were seeded in 6 well plates at a concentration of 2.4x10^5^ cells/well overnight. The seeded cells were treated with the desired concentrations of flavokawain A for 24, 48 and 72 hours. After the designated incubation time, the treated cells were collected and harvested according to the desired time points. The resulting pellets were immediately resuspended in the provided binding buffer and subsequently stained with 5 µl of FITC Annexin V and 5 µl of PI. The mixture was left to incubate at room temperature for 15 minutes. Afterwards, the cells were analyzed using the FACS system (Becton Dickinson, USA).

### JC-1 (MitoScreen) Assay

The BD MitoScreen Kit was used (Becton Dickinson, Franklin Lakes, NJ, USA) to measure the depolarization of the mitochondrial membrane potential. The cells were seeded in 6 well plates at a density of 2.4x10^5^ cells/ml. The following day, the cells were treated with three different concentrations of flavokawain A and incubated in a humidified CO_2_ incubator. After 48 hours, the cells were collected by trypsinization and centrifuged at 2000 rpm for 5 minutes. Around 1x10^6^ cells were incubated with 500 µl of JC-1 working solution. The JC-1 working solution contains the JC-1 stock solution and assay buffer at a 1∶100 ratio. This mixture was incubated at 37°C for 15 minutes. Afterwards, the cells were washed with the assay buffer twice, before proceeding to the FACS analysis (Becton Dickinson, USA).

### Caspase 8/9 FluorometricDetection Assay

To determine the activation of Caspase 8/9 we used the CaspGLOW Red Active Caspase-8/9 Staining Kit (BioVision Inc, USA). The cells were seeded in 6 well plates at a density of 2.4x10^5^ cells/ml. The following day, the cells were treated with three different concentrations of flavokawain A for 48 hours. Next, the cells were harvested by trypsinization and centrifuged at 2000 rpm for 5 minutes. 1 µl of Red-IETD-FMK (Caspase-8)/Red-LEHD-FMK (Caspase-9) were added to 1x10^6^ cells and incubated at 37°C for 30 minutes. Subsequently, the cells were washed twice before being subjected to FACS analysis (Becton Dickinson, USA).

### Wound Healing Assay

This assay was done using the protocol outlined by Liang et al [16]. MDA-MB231 cells were seeded to full confluency in 6 well plates over night. The following day, a scratch was introduced in the middle of the well using a sterile yellow tip. The media was discarded and replaced with fresh media containing different concentrations of flavokawain A. The rate of migration towards the center of the wound was captured every three hours up until 24 hours (Nikon, Japan). The following formula was used to calculate the rate of migration: 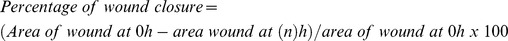



### *In vitro* Transwell Migration/Invasion Assay

The transwell migration/invasion assay was attempted based on the predicament that MDA-MB231 cells are able to migrate/invade with the presence of stimulants. This assay was conducted based on the protocol by Chen (2005) [17]. In the invasion assay, a layer of matrigel was applied to the upper chamber, whereas for the migration assay, the chamber was not coated with any basement membrane. This extracellular basement membrane was prepared by diluting to a ratio of 1∶3 with serum-free media and was left to solidify for 2 hours in a 37°C incubator. Prior to the experiment, MDA-MB231 cells were serum starved for 24 hours before being seeded in transwell chambers at a density of 2x10^5^ cells/ml on top of the solidified matrigel. In the lower compartment of the chamber, 2 ml of media supplemented with 10% FBS, 1 ml of conditioned 3T3 media and the desired concentrations of flavokawain A were added. The inserts were incubated in a 37°C CO_2_ incubator for 24 hours. Afterwards, the non-migrated/invaded cells were removed from the upper chamber of the transwell using a cotton swab. Migrated/invaded cells from the lower part of the membrane were fixed in methanol for 30 minutes before being stained with 0.3% of crystal violet. The membranes were later photographed using an inverted microscope and analyzed (Nikon, Japan).

### *In vitro* HUVEC Tube Formation Assay

The HUVEC tube formation assay was performed as summarized by Ponce, 2009 [18]. Firstly, 50 µl of undiluted matrigel was layered in a 96 well plate for 2 hours. The HUVEC cells were trypsinized and washed with PBS three times before being added to the precoated 96 well plates at a density of 1x10^5^ cells/ml together with the flavokawain A treatment. The plate was then incubated in a 37°C humidified CO_2_ incubator for 18 hours. After the desired time incubation, the tubes were photographed under an inverted light microscope (Nikon, Japan).

### *Ex vivo* Rat Aortic Ring Assay

The dorsal aorta was isolated from 5–7 weeks of male Sprague-Drawley rats. The aorta was rinsed under sterile conditions with ice cold PBS three times before being cut into ∼1–1.5 mm pieces. The sections of the aorta were placed in a matrigel-precoated 96 well plate. Afterwards, another layer of matrigel (50 µl) was placed on top of the aorta sections to sandwich it in between. Once the top layer of matrigel has solidified, fresh EGM media with the addition of flavokawain A were added to the wells. The aorta was left in a 37°C-humidified incubator for 7 days before being photographed and analyzed.

### Quantitative Real Time PCR Assay

Total RNA was isolated by using the QIAGEN RNaeasy Kit according to the manufacturer's protocol (Qiagen, Germany). The purity and concentration of the isolated RNA were measured using a spectrophotometer (Beckman Coulter) and the integrity of the RNA was determined by running a standard agarose gel. Then, 1 µg of the RNA was converted to cDNA using the QuantiTect Reverse Trasncription Kit according to the manufacturer's protocol (Qiagen, Germany). Afterwards, real-time PCR was carried out using the Power SYBR Green PCR Master Mix (Invitrogen, Carlsbad, CA, USA) on the iCycler IQ5 (Bio-Rad, USA). The accession number and sequence of the selected genes is illustrated in table 2.

**Table 2:** The accession number and sequence of the primers used in the quantitative real-time PCR assay.

Accession Number	Gene	Sequence
NM_001101.3	ACTB	F: 5′-AGAGCTACGAGCTGCCTGAC-3′R: 5′-AGCACTGTGTTGGCGTACAG-3′
NM_002046.4	GAPDH	F: 5- GGATTTGGTCGTATTGGGC-3R: 5- TGGAAGATGGTGATGGGATT-3
HQ387008.1	18S rRNA	F: 5- GTAACCCGTTGAACCCCATT-3R: 5- CCATCCAATCGGTAGTAGCG -3
NM_001220778.1	p21	F: 5- TGTCCGTCAGAACCCATGC-3R: 5- AAAGTCGAAGTTCCATCGCTC-3
NM_005030.3	PLK1	F: 5-CCTGCACCGAAACCGAGTTAT-3R: 5- CCGTCATATTCGACTTTGGTTGC-3
NM_004064.3	p27	F: 5-TCCGGCTAACTCTGAGGACA-3R: 5-AAGAATCGTCGGTTGCAGGT-3
NM_001243089.1	FOXM1	F: 5-ATACGTGGATTGAGGACCACT-3R: 5-TCCAATGTCAAGTAGCGGTTG -3
NM_006516.2	GLUT1	F: 5-ACAACACTGGAGTCATCAATGC-3R: 5-CCACAGAGAAGGAGCCAATCA-3
NM_000201.2	ICAM	F: 5-GACCCCAACCCTTGATGATA-3R: 5-GTGCTTTTGTGCCGATAGAA-3
NM_001287044.1	VEGF	F: 5-GCTGTGGACTTGAGTTGGG-3R: 5-GCTGGGTTTGTCGGTGTT-3

### Western Blot

Total protein lysates were obtained by lysing the cells with RIPA buffer supplemented with phosphatase inhibitor cocktail (Roche, Canada). The protein content was then measured by using the Bradford assay (Sigma, USA). Then, 100 µg of each sample were subjected to a 10% SDS-Page. The proteins were then transferred to a PVDF membrane (Roche, Laval, Canada) using the Pierce Fast Semi-Dry Blotter (Pierce, USA). Afterwards, the membrane was blocked with 0.5% skimmed milk overnight. The following day, the membranes were washed in TBST for 3 times and incubated with the designated antibodies, anti-BCL2 (ab18210, Abcam, USA), anti-cytochrome c (ab13575, Abcam, USA), anti-p27 kip1 (ab32034, Abcam, USA), Afterwards, the membranes were incubated in the appropriate secondary antibodies conjugated with HRP. The western blots were then developed under chemiluminescence condition (SuperSignal West Pico, Pierce, USA) using the ChemiDoc XRS machine (Bio-rad, USA). The bands were then analyzed using the Quantity One 1D Analysis software (Bio-rad, USA).

### Statistical Analysis

The data are presented as statistical means ± S.E (Standard Errors). The cut-off p value for significance was set at p< 0.05. The statistical comparison analysis was done using the one-way ANOVA. Graphpad Prism version 4 was used to perform all statistical analysis.

## Results

### Synthesis of FlavokawainA (E)-1-(2'-Hydroxy-4',6'-dimethoxyphenyl)-3-(4-methoxyphenylprop-2-en-1-one

Yellow flat crystals: Yield 72.6%, mp 112-115 °C. (Molecular formula C_18_H_18_O_5_).^1^HNMR (CDCl_3_, 500 MHz). δ 12.2 (s, 1H, OH), 8.10(d, 1H, *J*  =  15.5 Hz, Hβ), 7.79 (d, 1H, *J* =  15.5 Hz, Hα), 7.70 (brd, 2H, H-2, 6), 7.48 (m, 2H, H-3, 5), 6.00 (br,s, 1H, H-3'), 6.79 (br, s, 1H, H-5'), 3.89 (s, 3H, OMe, C-6'), 3.86 (s, 3H, OMe, C-4'), 3.80 (s, 3H, OMe, C-4). EI-MS *m/z* 298.0.

### Flavokawain A inhibits the proliferation of MCF-7 and MDA-MB231

**Figure 1:**
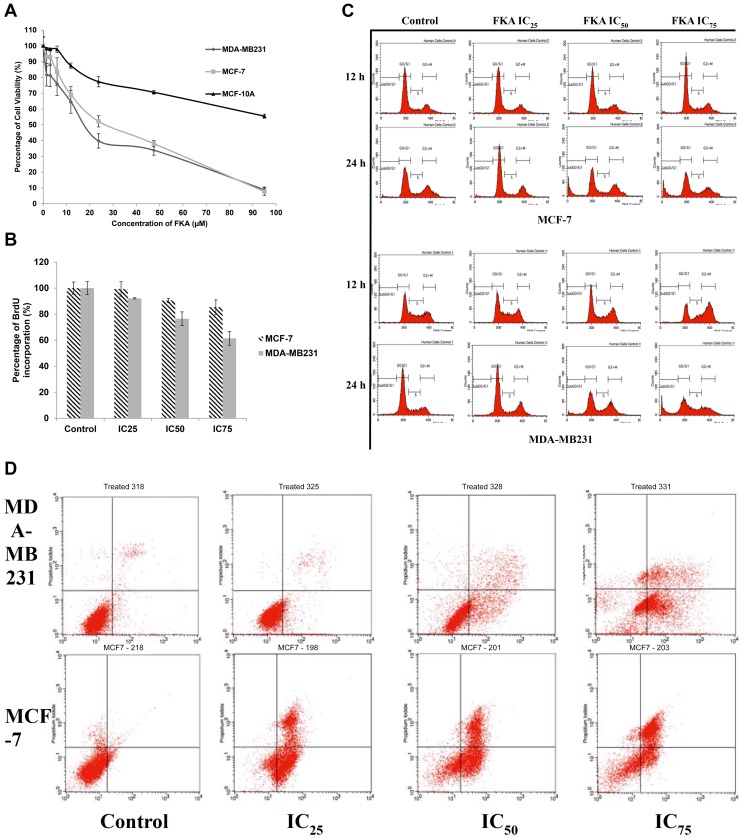
Flavokawain A inhibits the proliferation and induces apoptosis in both MCF-7 and MDA-MB231. A) The percentage of cell viability of MDA-MB231, MCF-7 and MCF-10A when treated with Flavokawain A for 72 hours.B) The percentage of BrdU incorporation in MCF-7 and MDA-MB231 after being treated with three different concentrations of flavokawain A for 48 hours.C) Histogram analysis of the cell cycle machinery in MCF-7 and MDA-MB231 after being treated with three different doses of flavokawain A for 12 and 24 hours.D) Histogram analysis of Annexin V/FITC in MDA-MB231 and MCF-7 after being treated with three different concentrations of flavokawain A after 48 hours. Data are expressed as mean ± S.E.M with p value< 0.05.

To assess the anti-proliferative effect of flavokawain A on both MCF-7 and MDA-MB231, as well as on the non-transformed mammary epithelial cell line, MCF-10A, the MTT assay was conducted. The cells were treated with 2-fold serial dilutions of Flavokawain A for 72 hours. Tamoxifen served as a positive control in this assay. Based on Figure 1A, the effects of flavokawain A is dose-dependent with 50% of the cell viability is suppressed below 50 µM. The IC_50_, (half-maximal inhibitory concentration) of flavokawain A is lower in MDA-MB231 (17.49 µM) than MCF-7 (25.13 µM) according to Table 3. The selectivity index was calculated based on the ratio of the IC_50_ obtained. In Table 3, it can be seen that the selectivity index of flavokawain A is substantially higher than tamoxifen in both MCF-7 and MDA-MB231. The BrdU incorporation assay was also done to confirm the anti-proliferative effects of flavokawain A (FKA). As seen in Figure 1B, the percentage of BrdU incorporation decreases as the dose of FKA elevates. Nonetheless, it is evident that FKA is more potent in inhibiting the proliferation of MDA-MB231 than MCF-7.

**Table 3:** The average values of IC_50_ in MCF-7, MDA-MB231 and MCF-10A after being treated with FKA and tamoxifen, as well as the selectivity index.

Cell Lines	Flavokawain A	Tamoxifen
	IC_50_ (µM)	
MDA-MB231	17.49 ± 1.50	25.84 ± 1.60
MCF7	25.13 ± 3.00	24.76 ± 0.80
MCF-10A	>100	26.24 ± 1.21
Selectivity Index		
MCF-10A/MDA-MB231	5.42	1.01
MCF-10A/MCF-7	3.78	1.06

### Cell cycle accumulation at the G2/M phase in MDA-MB231

To further examine the effects of FKA on the induction of apoptosis, the effects on the cell cycle machinery was investigated. Comparing to the control group, there was a substantial increase in the percentage of cells at the G2/M phase as the dose of FKA increased from IC_25_ to IC_50_ to IC_75_in MDA-MB231 cells. As in Figure 1C and Figure S1, the percentage of cells in the G2/M phase escalated from 24.89±0.25% in the control group to 54.96±0.57% in the IC_75_ treatment group. After 24 hours of treatment, in the MDA-MB231 cells, the percentage of SubG0/G1 cells increased slowly as the concentration of FKA increases. The percentage of cells in the G2/M phase also increased marginally from 16.86±1.07% to 25.63±0.41% in the IC_25_ group, 26.83±0.40% in the IC_50_ group and 23.93±0.46% in the IC_75_ treatment group. Interestingly, there were no significant arrests at any phase after 12 hours of treatment in MCF-7. Nevertheless, after 24 hours of treatment in MCF-7, there was a slight arrest at the G1 phase in the IC_25_ treatment group from 55.44±0.54% to 66.83±0.72%. The percentage of Sub G0/G1 cells in MCF-7 also increased as the concentration of FKA escalates.

### Flavokawain A induces apoptosis in MCF-7 and MDA-MB231

It is imperative to assess the mode of cell death that FKA induces. The morphological changes of both MCF-7 and MDA-MB231 upon treatment with flavokawain A were also observed via AO/PI double staining (Figure S2). The detection of the externalization of phosphatidylserine was performed. Based on Figure 1D, there is a pattern of cell population shifting from viable to early apoptosis to late apoptosis/necrosis in both MCF-7 and MDA-MB231. The percentage of early apoptotic cells in MDA-MB231 increased gradually from 4% in the control group up until 32% in IC_75_ of the treatment group. A similar pattern can be seen in the late apoptotic/necrotic cells as well, according to Figure 1D and figure S3. There is a direct proportional relationship between the percentage of apoptotic cells and the dose of FKA. In MCF-7 treated cells, the percentage of late apoptotic/necrotic cells also gradually increased from 1% (control) to 43% (IC_75_) as the dose escalates. This suggests that FKA is cytotoxic and induces apoptosis towards both cell lines in a dose-dependent manner, similar to the MTT results.

### Flavokawain Ainduces changes in the Mitochondrial Membrane Potential (ΔΨm)

**Figure 2:**
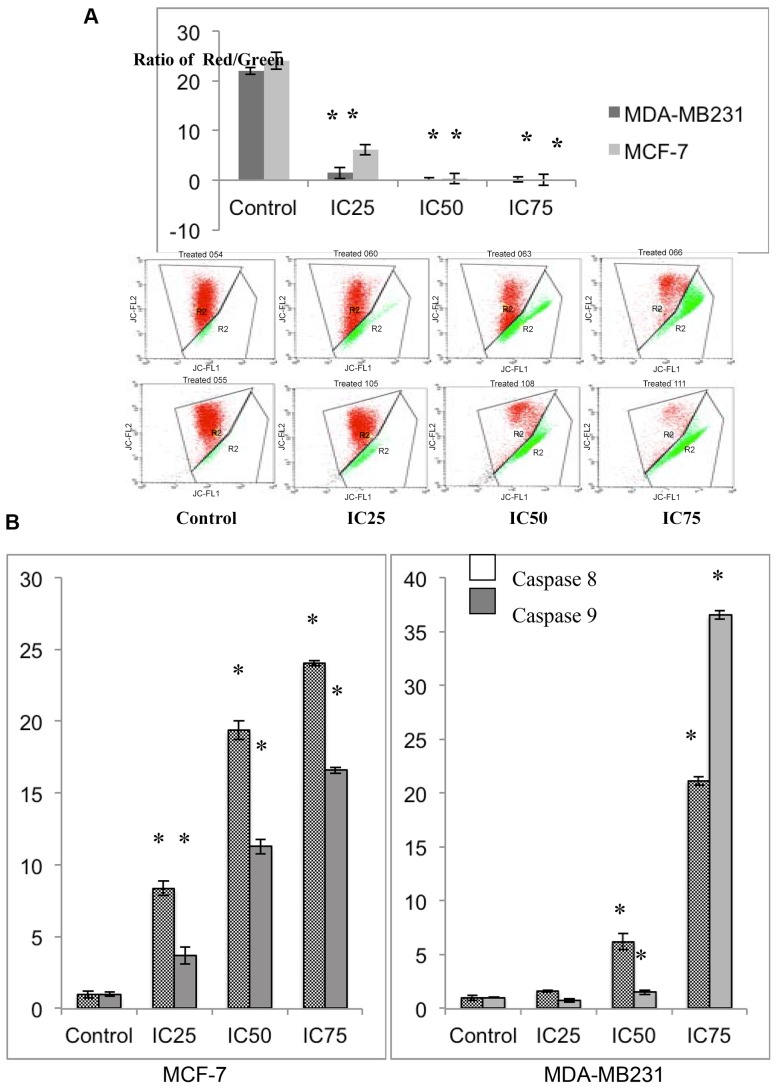
Flavokawain A induces changes in the mitochondrial membrane potential and activates caspase 8 and 9 in MCF-7 and MDA-MB231. A) Bar chart and histogram analysis of the depolarization of mitochondrial membrane potential of MCF-7 and MDA-MB231 after treatment with three doses of flavokawain A for 48 hours.B) Detection of the activation of caspase 8 and 9 in both MCF-7 and MDA-MB231 after 48 hours of treatment with three concentrations of flavokawain A. Data are expressed as mean ± S.E.M..(p<0.05)

To measure the changes in the mitochondrial membrane potential in MCF-7 and MDA-MB231 cells when treated with FKA, the JC-1 dye; that emits green fluorescence when it is in monomers and red when it aggregates, was used. The ratio of green fluorescence to red fluorescence is proportional to the strength of the mitochondrial membrane potential **(ΔΨm)** [19], [20]. Control, healthy cells usually have a polarized **(ΔΨm)** and can be detected as aggregates (red fluorescence) (Figure 2A). In FKA-treated cells, there is a shift in between the percentage of monomers and aggregates as the dose is increased in both cell lines, MCF-7 and MDA-MB231. The higher the dose of the FKA treatment, the lower the ratio of monomers to aggregates as depicted in Figure 2A.

### FKA mediates apoptosis through caspase 8 and 9

To determine the mode of apoptosis FKA was inducing, the activation of caspases 8 and 9 in both MCF-7 and MDA-MB231 was determined. After 48 hours of treatment with three different concentrations of FKA, there is a staggered increase in the activation of both caspase 8 and 9 in MCF-7 depending on the dose given. A seen in Figure 2B, it can be implied that the effects of FKA on MCF-7 is dose-dependent. Interestingly, there is a much more significant increase in caspase 8 than caspase 9. Similarly, the same pattern can be seen in MDA-MB231 treated cells, the level of activation of caspase 8 and 9 is depending on the dose given. Nevertheless, unlike MCF-7 treated cells, there is a much more significant increase in the activation of caspase 9 than caspase 8 in MDA-MB231 cells.

### FKA inhibits the motility and invasiveness of MDA-MB231 cells i*n vitro*

**Figure 3:**
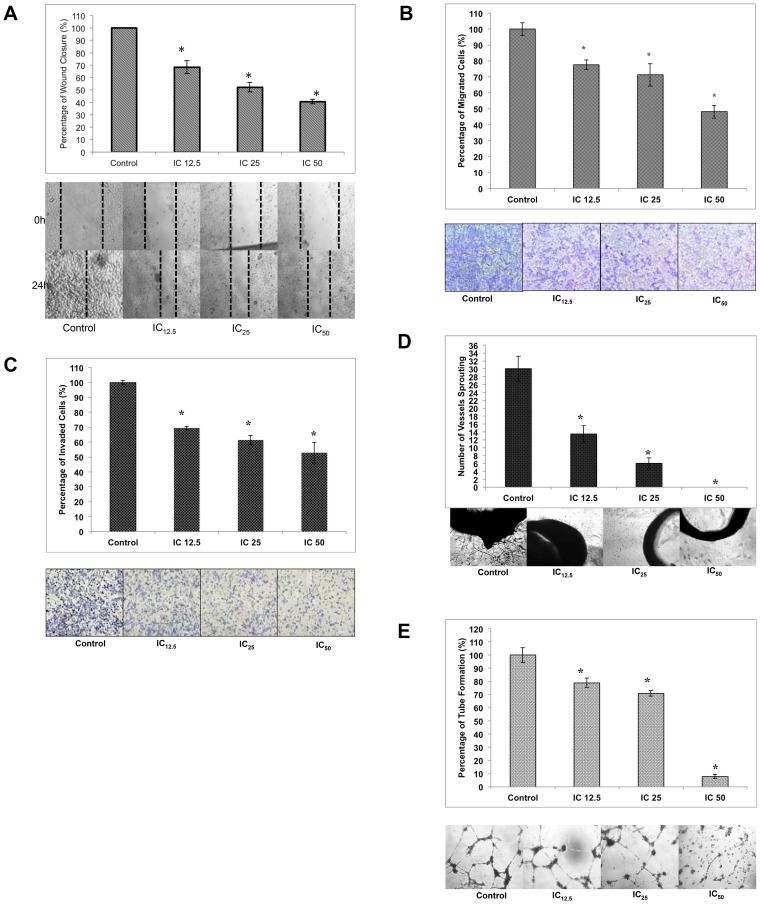
Flavokawain A inhibits the migration and invasion of cancer cells and possess anti-angiogenic properties. A) Percentage of wound closure in MDA-MB231 cells when a wound is introduced. Images represent the wound healing of MDA-MB231 when treated with three different doses of flavokawain A. Magnification: 10x. B) Images ofthe*in vitro*transwell migration analysis of MDA-MB231 when treated with three different doses of flavokawain A. for 24 hours. The cells were allowed to migrate through an 8 µM pore membrane. C)Images of the *in vitro* invasion analysis of MDA-MB231 when treated with three different doses of flavokawain A. The cells were allowed to invade through a layer of Matrigel for 24 hours. D) *In vitro* HUVEC tube formation analysis when treated with three different doses of flavokawain A for 18 hours. Magnification: 10X.E) *Ex vivo* rat aortic ring analysis when treated with three different doses of flavokawain A. The protrusions of vessels from the aorta were counted individually. The experiment was done in triplicates and the data are expressed as mean ± S.E.M. Magnification: 10X.

To further understand the anti-cancer activity of FKA, the wound-healing assay was conducted in accordance to Liang et al [16]. This assay was attempted in MDA-MB231 only since it is highly invasive than MCF-7. The rate of migration of MDA-MB231 cells is dose-dependent; the higher the dose of FKA given, the lower the percentage of migrated cells towards the center of the wound. In Figure 3A, it can be seen that in the IC_12.5_ treatment group, the percentage of wound closure decreased to 68.48±5.12% from 100% in the control group. There is an additional decrement in the IC_25_ and IC_50_ group with the percentage of wound closure, 52.29±3.85% and 40.61±1.85% respectively.

To further confirm the anti-migration effects of FKA, the transwell *in vitro* migration assay was performed. Based on Figure 3B, the rate of migration through the transwell membrane decreased as the dose is increased. The IC_12.5_ treatment group had a 77.64±3.5%; this percentage was reduced in the IC_25_ treatment group to 71.28±7.3%. Expectantly, the percentage of migrated cells in the IC_50_ group also declined. The percentage went down to 48.11±4.7%.

The invasiveness of MDA-MB231 through a layer of matrigel when treated with FKA was also measured. Similar to the *in vitro* migration assay, the *in vitro* invasion assay was also dose-dependent. In the lowest treatment group, IC_12.5_, the percentage of invaded cells was 69.55±1.16%. The percentage decreased to 61.48±3.0% in the IC_25_ group. As depicted in Figure 3C, the percentage of the IC_50_ group was 52.98±6.9%.

### FKA possesses anti-angiogenic potential

In part of the metastatic cascading steps, angiogenesis also plays a major role in the formation of secondary tumors. To further investigate the anti-metastatic potential of FKA, two angiogenic assays were performed. The HUVEC tube formation assay was conducted on a layer of matrigel. The number of tubes formed were scored and analyzed based on the dose of FKA given. As depicted in Figure 3D, there is a decline in the number of tubes formed as the dose of FKA is increased. This indicates that FKA inhibits the formation of new blood vessels *in vitro*.

To further confirm the anti-angiogenic potential of FKA, the *ex vivo* rat aortic ring assay was conducted. Similar to the HUVEC tube formation assay, in Figure 3E, the outgrowth of vessels from the fragmented aorta is impeded as the dose of FKA escalates. This implies that the anti-angiogenic potential of FKA is dose-dependent.

### FKA regulates several apoptosis and metastatic-related genes and proteins

**Figure 4:**
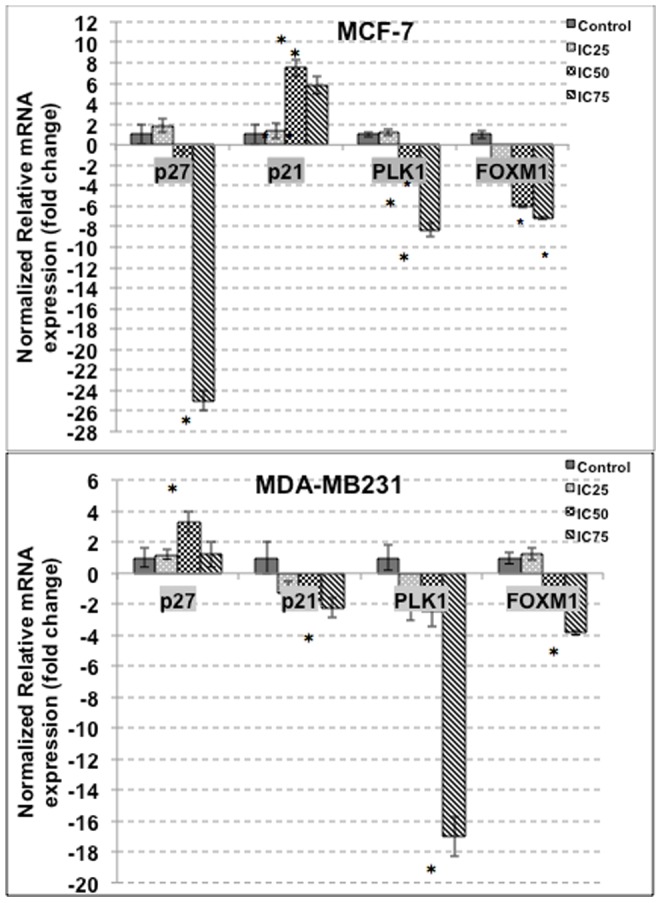
qPCR analysis of apoptosis and cell cycle related genes; p27, p21, PLK1 and WEE1 in MCF-7 and MDA-MB231 after treatment with three doses of flavokawain A for 18 hours. The experiment was done in triplicates and the data are expressed as mean ± S.E.M.

**Figure 5:**
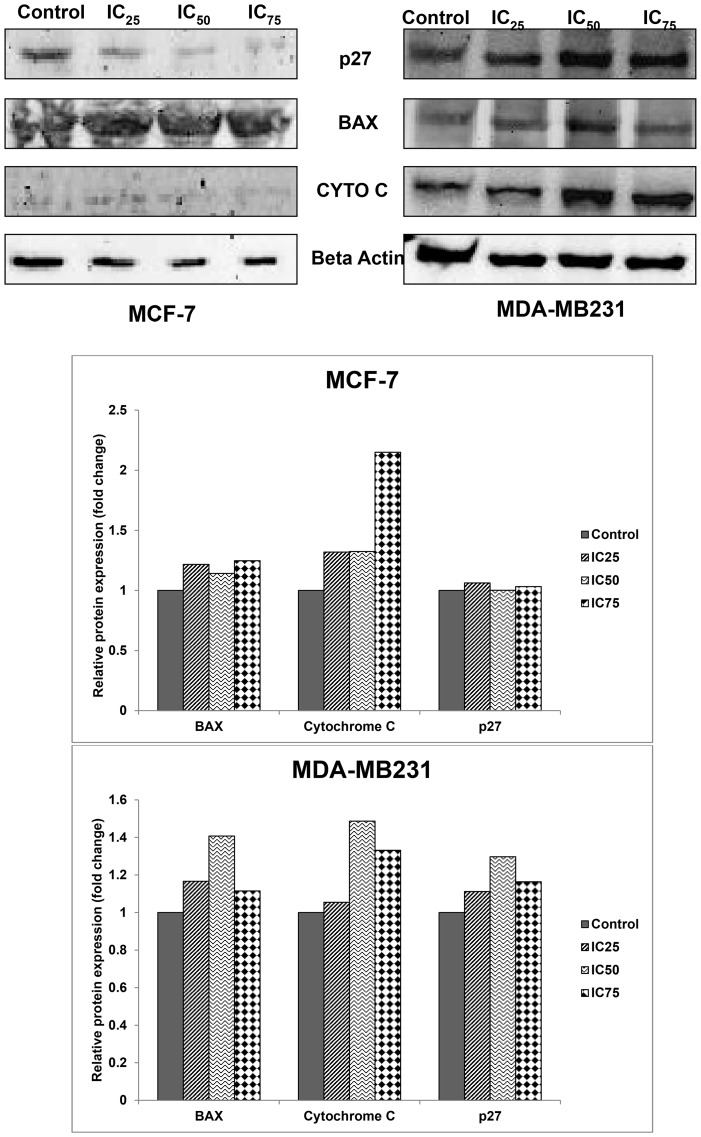
Western Blot analysis of apoptotic-related proteins; BAX, Cytochrome C, and p27 in both MCF-7 and MDA-MB231 after 24 hours of treatment with three concentrations of flavokawain A.

**Figure 6:**
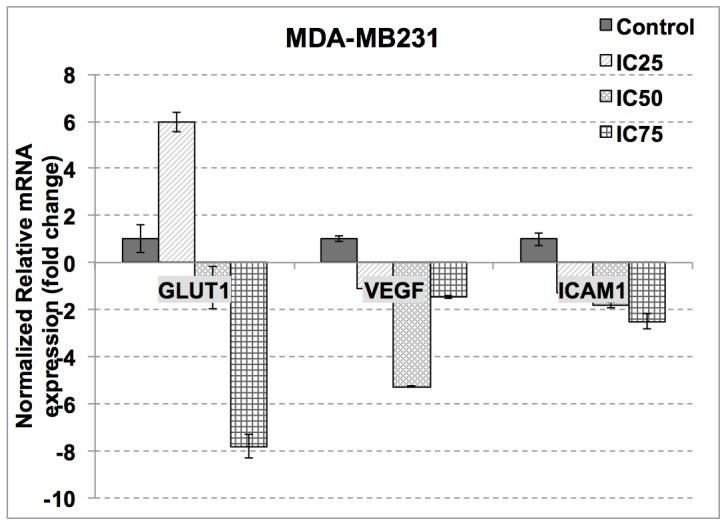
qPCR analysis of metastasis-related genes; GLUT1, VEGF and ICAM in Flavokawain A-treated MDA-MB231 after 18 hours of treatment. The experiment was done in triplicates and the data are expressed as mean ± S.E.M.

The effects of apoptotic related genes and proteins in MCF-7 and MDA-MB231 when treated with FKA were measured by qPCR and western blot. In MCF-7, the level of p27, PLK1 and FOXM1 decreased whereas the level of p21 increased in the treatment groups. As in Figure 4, in MDA-MB231 however, the level of p21, PLK1 and FOXM1 decreased, as the level p27 increased. The protein level of BAX and cytochrome c increased in both cell lines as shown in Figure 5. Nevertheless, the protein level of p27 declined in MCF-7 but was shown otherwise in MDA-MB231. For the metastatic genes, the expressions were only tested in MDA-MB231 treated cells only, the mRNA level of GLUT1, ICAM and VEGF decreased significantly as shown in Figure 6.

## Discussion

Fighting cancer has been an ongoing battle for decades, and even now, there is no exact way to treat this disease. One of the most recent approaches to treat breast cancer is by targeting the human endogenous retrovirus-k antigens using antibodies[21], [22]. Although this method is still preliminary it still holds promising development [21], [22]. Nevertheless, though drug therapy has been revolutionizing rapidly in the past decade, the options of selecting a viable drug is still limited.FKA has been reported to be cytotoxic towards bladder cancer [10]. Nevertheless, to the best of our knowledge, the effect of FKA on breast cancer *in vitro* has not been reported yet. The operating mechanism of FKA is not fully understood yet and therefore, this study attempts to elucidate the mechanism of cytotoxic and anti-metastasis effects.

As evidenced by the preliminary MTT assay, FKA inhibited the proliferation of both MCF-7 and MDA-MB231 in a dose dependent manner. Based on the IC_50_ value, FKA inhibited the proliferation in MDA-MB231 better than in MCF-7. In terms of selectivity, FKA is more selective than tamoxifen, a commonly used drug to treat breast cancer. The selectivity index of FKA in MDA-MB231 is much higher than in MCF-7. The results obtained from our study suggest that FKA was cytotoxic toward both MCF-7 and MDA-MB231. The IC_50_ values of FKA on two types of bladder cancer with different status of p53 were reported to be around 17 µM [9].As shown in our Annexin V FACS analysis, in FKA-treated cells, there is a shift in the pattern of the externalization of phosphatidylserine. This indicates that FKA induced apoptosis in both cell lines. Additionally, this perception was further confirmed based on the changes in the cell morphological features as seen in the double staining AO/PI. Furthermore, as demonstrated by the JC-1 assay, FKA triggered the depolarization in the mitochondrial membrane potential in both MCF-7 and MDA-MB231. To use JC-1 as a main indicator regarding the changes of the mitochondrial membrane potential is bias. Therefore, the mechanistic activity of FKA was further analyzed by the expression of apoptotic related proteins and genes. FKA increased the expression of BAX as well as cytochrome c in both MCF-7 and MDA-MB231. Bax is traditionally known as a member of the BCL family and is widely known as its role as a pro-apoptotic protein [23]. Bax is one of the proteins that induces changes in the mitochondrial potential upon apoptotic stimuli, this ultimately leads to the secretion of cytochrome c, another pro-apoptotic protein [23], [24]. Cytochrome c, in turns, stimulates the caspase cascade activation and subsequently, cell death [24]. The activation of caspase 9 is directly related to the level of cytochrome c. Caspase 9 is known to be involved in the activation of the intrinsic apoptosis pathway [25]. The activation of caspase 9 is essential to induce the changes in the mitochondrial morphological features as well as ROS production [25]. Caspase 8 on the other hand, is more likely to be involved in the execution of apoptosis through the extrinsic pathway [26]. This protein is usually activated by receptors such as Fas ligand or TNF receptors [27]. Downstream of caspase 8 is usually other effector caspases that induces apoptosis [27]. As depicted in Figure 3B, there is a substantial increase in the activation of caspase 8 and 9 in both MCF-7 and MDA-MB231. Interestingly, in MCF-7, the activation of caspase 8 is more significant than caspase 9, unlike MDA-MB231 where the reverse effect is seen. The level of cytochrome c is much more higher in MDA-MB231 than MCF-7 and this could explain the higher level of activation of caspase 9. This implies that FKA may act differently in different types of cancer cells.

To further understand the action of FKA, the effects of FKA on the cell cycle progression was analyzed. Intriguingly, there were some differential effects of FKA in both MCF-7 and MDA-MB231. It can be seen in the cell cycle analysis, that FKA induced a significant G2/M arrest in MDA-MB231 after 12 and 24 hours of treatment, but not in MCF-7. In MCF-7 there was a minor arrest at the G1 phase after 24 hours of treatment, but this phase is shifted towards the sub G0/G1 phase consequently. It has been reported, that FKA induces different effects depending on the status of the p53, in p53-mutant cells it is more likely to induce a G2/M arrest instead of G1 arrest in p53-wild type cells [10]. This could explain the different effects of FKA on MCF-7 and MDA-MB231, as MCF-7 possesses wild-type p53 whereas MDA-MB231 has a mutant type p53 [28]. Based on the qPCR and western blot analysis, the level of p27 is increased in MDA-MB231 while the mRNA expression of p21, decreased significantly in MDA-MB231. The pattern of expression of both of these cell cycle regulatory proteins could explain the resulting effects of the G2/M phase arrest seen in MDA-MB231 cells. Nevertheless, for the MCF-7 treated cells, there is an increase in the p27 expression only in the IC25 treatment group, doses higher than that led to a decrement of the protein. Inversely, there was an increase in the p21 mRNA expression in MCF-7 cells. Both these proteins are major players in the G1 phase and as such, could explain the G1 arrest in MCF-7. The mRNA expression of G2/M related cell cycle regulatory proteins such as PLK1 and Forkhead Box M1 were also measured. PLK1 is a mitotic polo like kinase that controls multiple stages of cell cycle entries [29]–[31]. PLK1 is often overexpressed in several tumors including breast cancer [31]. The depletion of PLK1 is associated with the arrest at the G2/M phase and consequently, apoptosis [30]. FOXM1 on the other hand, is a substrate for PLK1 in regulating the cell cycle machinery [29]. As depicted in Figure 4, FKA managed to inhibit the mRNA expression of both PLK1 and FOXM1 significantly in MCF-7 and MDA-MB231. Nevertheless, the inhibition was much more significant in MDA-MB231 as there was a substantial G2/M arrest in the treated group.

The anti-cancer activity of FKA was further evaluated for its anti-metastatic potential. There are several steps in metastasis; and migration is one of the crucial stages [4]. In the wound-healing assay, the rate of migration of the cells towards the center of the wound is dependent on the dose of FKA given. This indicates that FKA is more effective in inhibiting the migration of cells at a higher concentration. Additionally, this pattern can also be seen in the *in vitro* transwell migration and invasion assay. The potential use of FKA as anti-cancer agent is further strengthened by the anti-angiogenic assays. Angiogenesis is a process whereby cancer cells form new blood vessels to supply for nutrients [32]. This step is vital in order for cancer cells to become malignant. As demonstrated in the *in vitro* tube formation assay, and the *ex vivo* rat aortic ring assay, FKA managed to inhibit the formation of new vessels significantly. From the qPCR and western blot results, FKA inhibited the expression of VEGF in MDA-MB231. VEGF is a common associate to metastasis, especially in breast cancer. The level of VEGF is highly correlated with the initiation of angiogenesis. On a related note, cancer cells have an unusual high demand of energy in order to sustain. The Warburg effect is a popular theory in the participation of aerobic glycolysis in tumor [33]. GLUT1 is a glucose transporter that plays a major role in the glycolytic pathway and is often related to the malignant type of cancer [34], [35]. FKA treatment decreased the mRNA expression of GLUT1 in MDA-MB231. Additionally, ICAM-1 is a glycoprotein that is regularly involved in the immune response and tumorigenesis [36]. This protein is highly expressed in most malignant tumors [36]. Based on the qPCR reactions, FKA was found to reduce the mRNA expression of ICAM1 significantly.

## Conclusions

**Figure 7:**
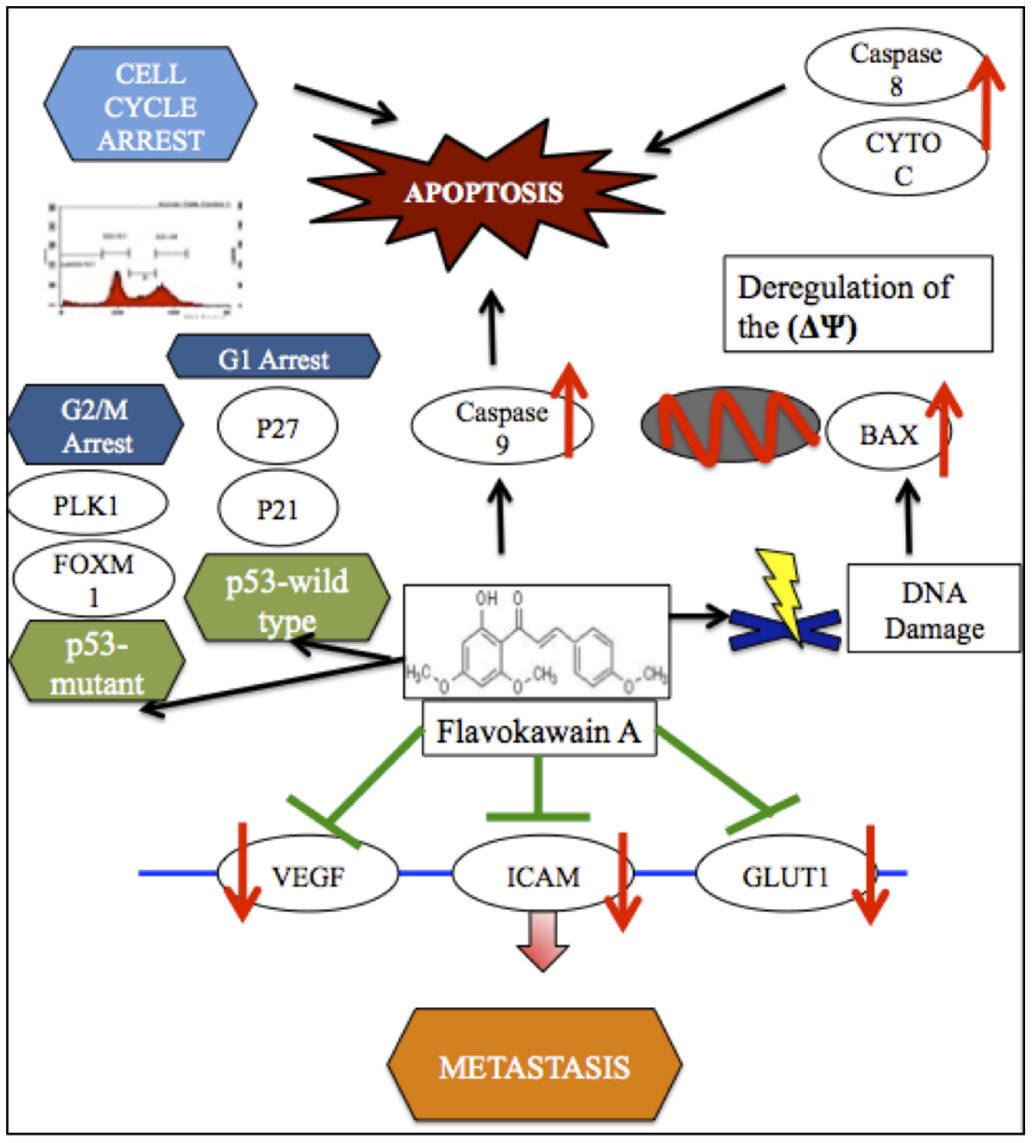
Probable operative mechanisms of Flavokawain A in cancer cells with different status of p53.

This study suggests that FKA has a promising anti-cancer potential especially in treating breast cancer. Figure 7 illustrates the possible pathway of FKA depending on the p53 status of the cancer cells. FKA induces apoptosis in both MCF-7 and MDA-MB231 dose-dependently. FKA induces G2/M arrest in MDA-MB231 only and thus implying that there is a selective anti-cancer activity of FKA depending on the p53 status. Thus, FKA is promising to treat more aggressive triple negative breast cancer with mutant p53.In terms of metastasis, FKA inhibited the migration and invasion of MDA-MB231 significantly. Additionally, FKA also holds promising anti-angiogenic potential as it impeded the growth of vessels in *in vitro* and *ex vivo*experimental models. Overall, FKA can be seen as a breakthrough candidate in battling cancer as well as become an anti-metastatic agent. Nevertheless, further in depth analysis including *in vivo* trial is needed to better understand the functional machinery of FKA.

## Supporting Information

Figure S1 **Bar chart analysis of the cell cycle analysis in MCF-7 and MDA-MB231 after 12 and 24 hours of treatment with flavokawain A.** The experiment was done in triplicates and the data are expressed as mean ± S.E.M. (p<0.05)
(TIFF)

Figure S2 **AO/PI double staining of MCF-7 and MDA-MB231 after being treated with three different doses of FKA for 48 and 72 hours.**
(TIFF)

Figure S3 **Bar chart analysis of the annexin v assay in MCF-7 and MDA-MB231 after 24 h, 48 h and 72 h of treatment with three doses of flavokawain A.** The experiment was done in triplicates and the data are expressed as mean ± S.E.M. (p<0.05)
(TIFF)
