# Supplementary figures and images for: Flavokawain A Induces Apoptosis in MCF-7 and MDA-MB231 and Inhibits the Metastatic Process In Vitro
Source: PLoS One. 2014 Oct 6;9(10):e105244. doi: 10.1371/journal.pone.0105244 (PMC4186755; doi:10.1371/journal.pone.0105244)

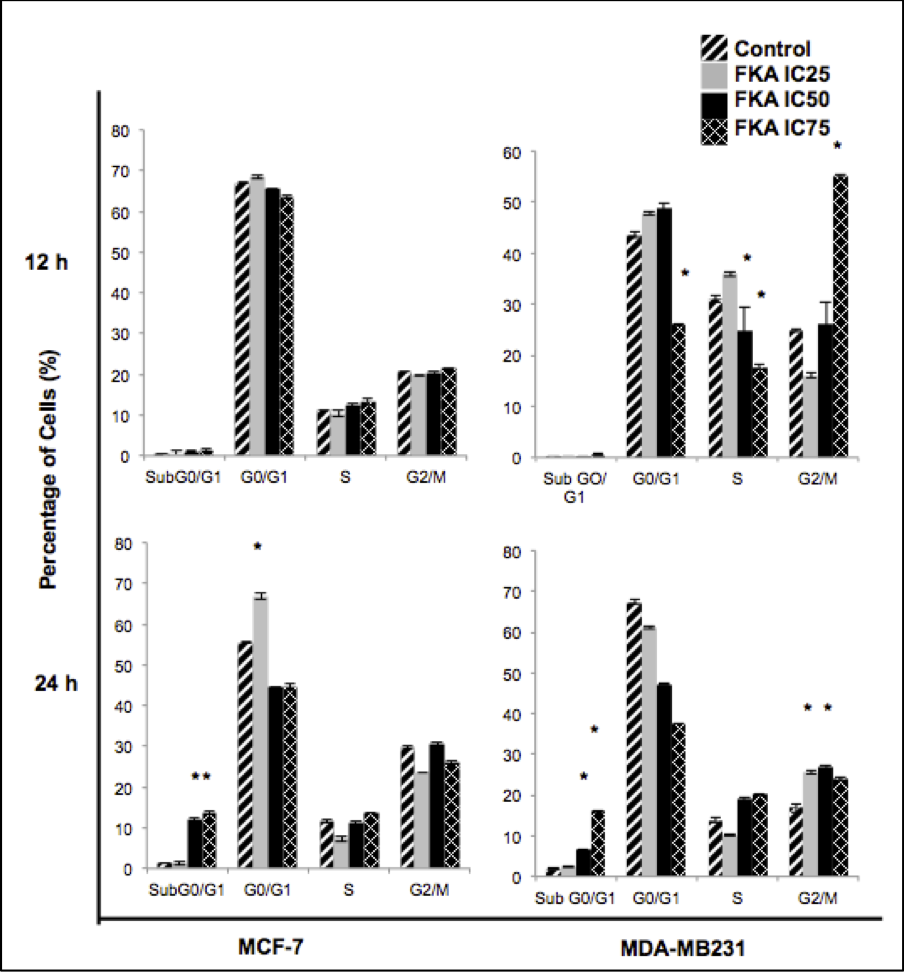

Supplement: Figure S1 — Bar chart analysis of the cell cycle analysis in MCF-7 and MDA-MB231 after 12 and 24 hours of treatment with flavokawain A. The experiment was done in triplicates and the data are expressed as mean ± S.E.M. (p<0.05) (TIFF) [file pone.0105244.s001.tiff]

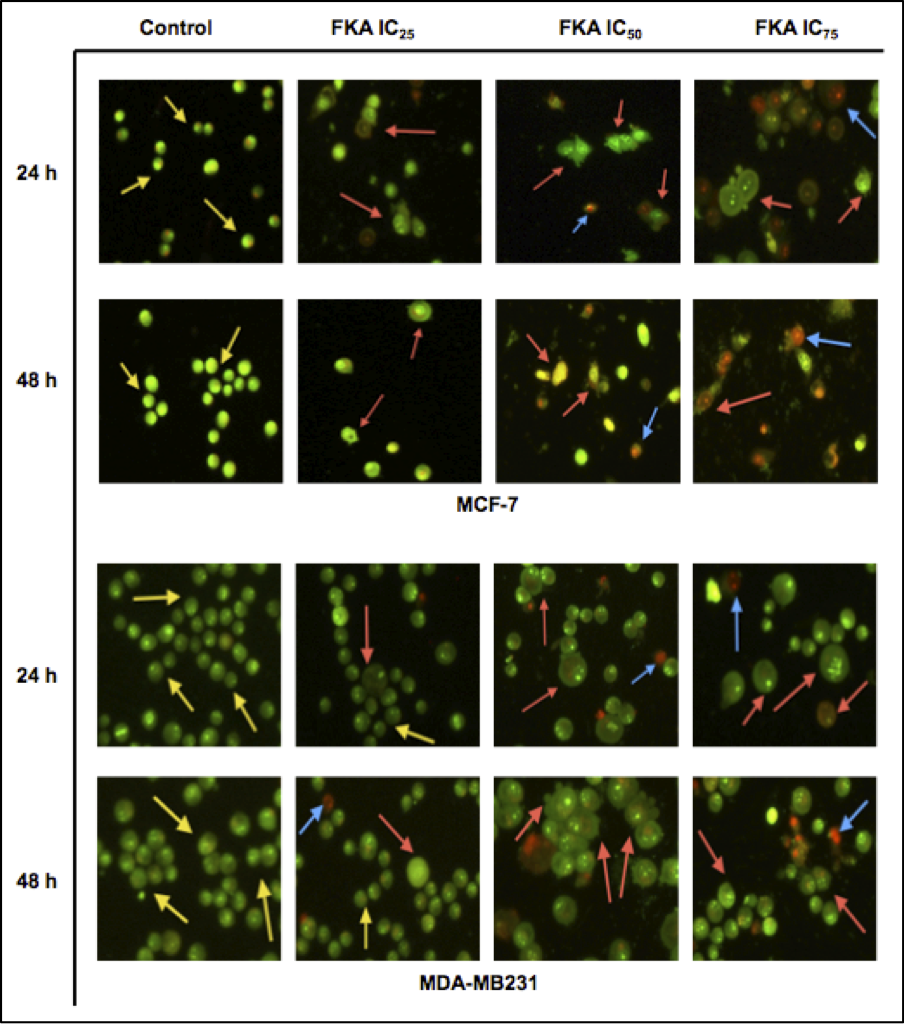

Supplement: Figure S2 — AO/PI double staining of MCF-7 and MDA-MB231 after being treated with three different doses of FKA for 48 and 72 hours. (TIFF) [file pone.0105244.s002.tiff]

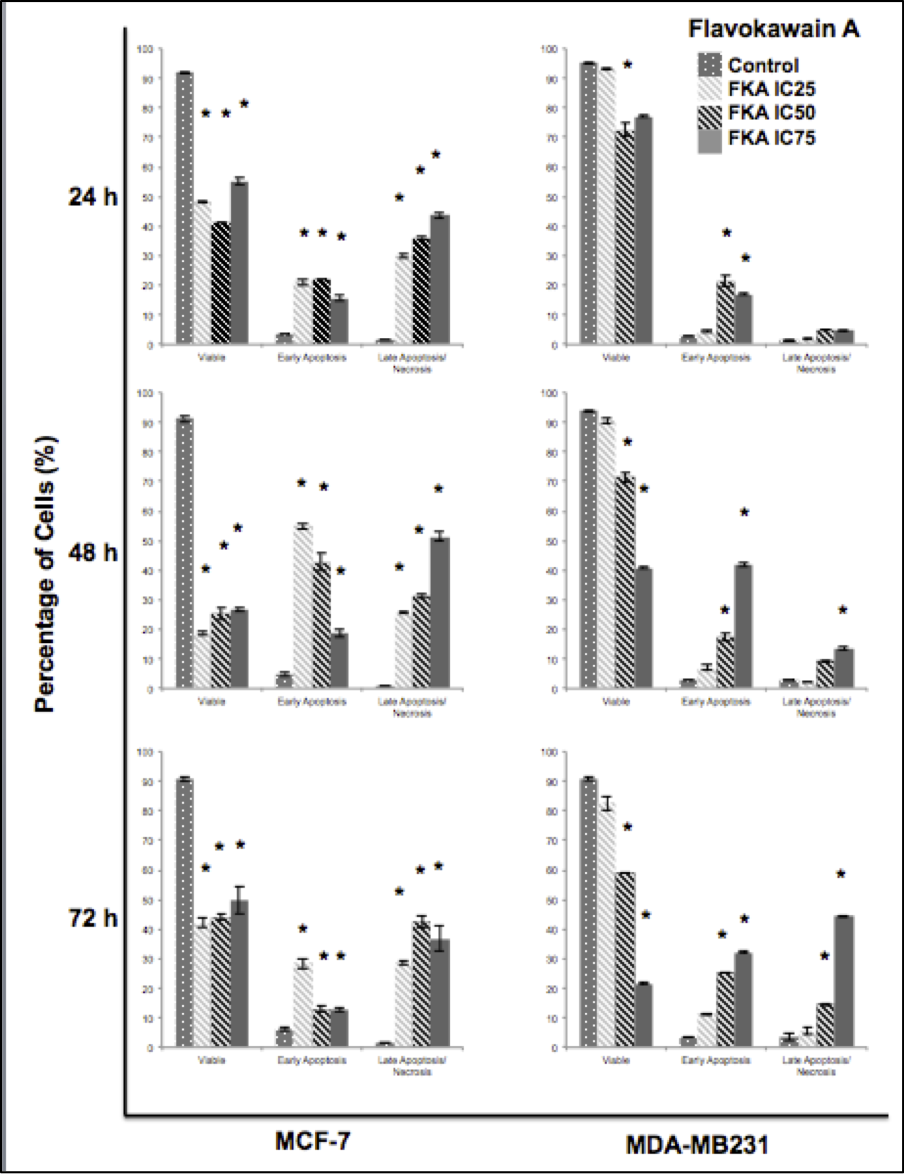

Supplement: Figure S3 — Bar chart analysis of the annexin v assay in MCF-7 and MDA-MB231 after 24 h, 48 h and 72 h of treatment with three doses of flavokawain A. The experiment was done in triplicates and the data are expressed as mean ± S.E.M. (p<0.05) (TIFF) [file pone.0105244.s003.tiff]
